# Chicago Medical Society—March, 1866

**Published:** 1866-04

**Authors:** 


### PROCEEDINGS OF MEDICAL SOCIETIES.

### CHICAGO MEDICAL SOCIETY—MARCH, 1866.

Dr. S. Wickersham reported a case of angular curvature of the spine in an infant which had been under his observation from birth, and which had apparently enjoyed perfect health during its first year. For the next six months, although taking an abundance of food, it ceased to grow, without, however, losing flesh. There was no diarrhœa, no vomiting, no cough, no pain, in fact no other evidence of sickness, except a degree of weakness in its lower extremities. The child did not at any time stand alone or make any attempt at walking. Upon an examination of its spine when it was about fifteen months old, no evidence of disease was found. At about the eighteenth month its mother thought she observed a slight swelling over its spine, and again requested Dr. W. to see it. There was then marked spinal disease, involving the second, third and fourth dorsal vertebræ, although the angular curvature was not extensive. The mother was *positive* that it had not fallen or received any injury. Believing that the disease was constitutional in its origin, and that scrofulosis and tuberculosis were identical, he directed the administration of such medicines as have received the sanction of many of the best authorities in the profession, cod liver oil being the chief one.

At about the twenty-first month it commenced to cough, and lose flesh; the angular curvature had become more prominent; there was dullness in the infra-clavicular region of the left side. Some weeks later a cavity was formed in the upper portion of the left lung, and undoubted evidence that the right lung was participating in the disease. At about the twenty-sixth month the hands and feet became dropsical. The child was greatly emaciated, but had at no time any diarrhœa, and took eagerly an abundance of nutritious food. It went from bad to worse, and died, aged twenty-seven months.

This case was brought to the attention of the members of the society, because a few weeks ago Prof. Bauer, of New York, delivered before the society a lecture, upon coxalgia and spinal disease, claiming in a very decided manner that they were generally the result of traumatic causes, and that the profession had heretofore entertained erroneous views in regard to their pathology, and also deprecating the use of internal remedies. That such diseases do sometimes result from blows, falls, etc., he was prepared to admit, but that the great majority of them result from constitutional causes, he should continue to think until more evidence was produced to the contrary.

No autopsy on this case was permitted; but that the child died from consumption is certain, or else rational and physical signs furnish us no knowledge of changes in the lung tissue.

Dr. McAllister related briefly the history of a patient, 17 years of age, now under his care in advanced stages of tuberculosis, who had recently suffered from coxalgia, as shown by the pain, contraction of the muscles and position of the limb, and by the crepitation readily perceived, when the patient was placed under the influence of chloroform. He considered the disease of the hip-joint as resulting wholly from tuberculosis.

In a discussion which followed the reports, Drs. Marguerat and Holmes maintained that the cases afforded but negative evidence. There was reason to believe that the infant might have received an injury, and that the resulting abscesses might have invaded the tissue of the lungs, producing all the symptoms described.

The case of Dr. McAllister might be an exceptional one.

Weak, ill-developed children were liable to injuries of the hip and vertebræ, which might possibly result in inflammation, even more readily than in healthy subjects. Strong, robust children were perhaps more liable to injury, on account of their activity in climbing and jumping. Such children, on receiving an injury, continue to exercise, thus inducing irritation of the affected parts. The subsequent pain, want of sleep, exercise and loss of appetite, with the irritative fever and suppuration, all long continued, were sufficient to produce the peculiar cachexia of these cases.

The fact that suitable local treatment, counter-extension and support of the parts, evacuation of the purulent matter, with care as regards ventilation and diet, result so very often not only in recovery from the local disease, but also in perfect restoration to health, would seem to prove that there was much less frequently than formerly supposed, a peculiar scrofulous or tuberculous diathesis in this class of cases.

Recent investigations with the microscope seemed to indicate that in all bones collected in museums, and supposed to contain tuberculous deposit, the abnormal product was simply inspissated pus; in fact, that there was no such substance as "tuberculous deposit," distinguishable with the microscope, from modified pus.

Dr. Tucker related the history of the following remarkable case:

He was called to a patient, who had been delivered some days before his visit with the aid of forceps. He found, on examination, an immense abscess at one side of the anus, extending upwards by the side of the rectum, the external tissues appearing ready to slough. The abscess, on being opened, discharged very large quantities of offensive pus. After the gangrenous portions had been thrown off, an extensive sinus was discovered by the side of the rectum, sufficiently large to almost admit the hand. This sinus terminated three or four inches above the anus, in a small opening into the rectum. There was also quite a large recto-vaginal fistula, near the upper end of the abscess. After dividing the septum between the rectum and abscess, as in ordinary fistula in ano, cicatrization slowly progressed and in two months was complete. The patient was then able to retain the fæces prefectly. It is a fact worthy of notice that the recto-vaginal fistula healed spontaneously.

Dr. Lyman related the case of a woman who was placed under his care in the Bellevue Hospital, New York. She had been delivered with forceps, by parties outside of the Hospital, and had suffered a rupture of the perineum and of the recto-vaginal septum, extending more than two-thirds of the distance between the anus and the fornix vaginæ. When brought into the Hospital, her condition was such as to preclude immediate operation, so she was simply placed in bed, with good diet, tonics and strict attention to cleanliness. At the end of a fortnight the rent in the recto-vaginal septum had closed as far down as the sphincter muscle. She was afterwards operated upon by paring the margins of the wound, and bringing them together with silver wire. After two such operations she was able to retain the fæces, and they passed through the anus when leaving the bowel. Feeling well and comfortable, she concluded to postpone operation upon the perineum, and left the Hospital.

In answer to a question, Dr. Fisher stated that he had observed two cases in which recto-vaginal fistula closed spontaneously.

Dr. Fisher presented a most interesting specimen of the uterus, ovaries and fallopian tubes, taken from a woman who had died from the effects of tubal pregnancy. The patient, in her fifth pregnancy, was suddenly and without warning seized with most violent pains in the abdomen, with great prostration, and intense desire to evacuate the contents of the bladder and rectum. Although opiates and stimulants were freely administered, the patient rapidly sank and died in eight and a half hours.

The autopsy revealed the rupture of a small artery, from which had been discharged into the peritoneal cavity eight pints of blood, nearly one-half of which was coagulated. In the left tube was found a fœtus about one inch and a half in length. The uterus was of its ordinary unimpregnated size, and was not lined with the membranes of pregnancy.

Dr. Marguerat exhibited a brain taken from a girl at the orphan asylum, eight years of age, who had died quite suddenly with the following symptoms:

The patient was of a scrofulous diathesis and subject to eruptions. Early on the morning of Tuesday, while in her usual health, she was suddenly attacked with vomiting, which continued during the day; the fluids from the stomach, as the attendant stated, being mixed with blood. No physician was called during the day. At his visit in the evening, Dr. M. found the pupil dilated, pulse 100, bowels very constipated. The patient was very restless, constantly tearing the tongue, gums and lips with the fingers to such an extent as to cause them to bleed freely.

On the next morning the pulse was 60, pupils contracted. Although there had been during the night slight spasms of the muscles of the neck, they had entirely subsided; the breathing was labored; the patient died comatose twenty-two hours after the commencement of the attack.

The brain simply showed considerable venous congestion, with slightly increased quantity of the fluids in the ventricles.

Dr. Adolphus stated that he had treated a case of asthenic puerperal fever, with ten grains of sulphate of lime every three hours, with no good effect.

Dr. Wanzer read a paper on the geology, climate and diseases of Santa Clara Valley, California, where he had recently passed several months.

Dr. Holmes exhibited a quite rare specimen of tumor of the iris, with the following history:

The patient from whom it was taken, a German woman, 26 years of age, observed eighteen months since a minute white spot in the temporal side of the left iris, half way between its pupillary edge and periphery. There was no pain, congestion, loss of sight, nor even any inconvenience whatever for several months. About ten months ago the tumor had gradually encroached upon the pupil, although there was no diminution of vision and no vascularity. The iris, except at the seat of disease, had been normal in color. At this period the tumor commenced to be subject to attacks of hemorrhage. The anterior chamber during these attacks would be quite suddenly and completely filled with blood, which entirely obstructed vision. The coagulum would be absorbed in a few days, when vision became again perfect. During every attack of hemorrhage there was considerable pain and congestion of the conjunctiva. This hemorrhage occurred without any known cause, either as connected with exercise, position, straining at stool, or with the monthly periods.

On applying for advice about a fortnight since, she was recovering from an attack of hemorrhage, which had caused much greater pain than ever before, still continuing very severe in and about the eye. The tumor had in three weeks doubled its size.

The conjunctiva and subjunctival membrane were much congested, especially near the cornea and at the temporal side of the globe; the tumor covered the pupil and filled the externa two-thirds of the anterior chamber, but did not quite extend to its upper and lower limits. The remaining portion of the anterior chamber was filled with coagulum, which was, however, evidently being absorbed. The tumor pressed against the cornea and appeared to extend posteriorly, as if involving the ciliary muscle; it was of a pinkish, white lustre, and presented a structure of great delicacy, resembling the malignant growths not unfrequently observed in the fundus of the eye, in children. The cornea was slightly ulcerated or abraded in front of the tumor.

The general appearance of the growth and the hemorrhage, would lead one to suspect it was of malignant nature, although no evidence of family predisposition could be discovered. Dr. D. Prince, of Springfield, who happened to be present at the examination, concurred in this suspicion.

The size of the tumor, the indications that it extended to the ciliary muscle, and the ulceration of the cornea, almost wholly precluded the feasibility of extracting it with its attachments, even if non-malignancy could be established. It was thought best therefore to extirpate the globe by enucleation.

The specimen here presented consists of the globe divided horizontally through its centre. The internal structure of the tumor with its encroachment upon the ciliary muscle are readily seen. Dr. W. C. Hunt, after a careful microscopic examination of the tumor, describes its structure as follows:

Irregularly shaped cells, of which the caudate form predominates, filled with nuclei and nucleoli.

Large quantities of nuclei floating loose.

In some of the cells, and also with the free nuclei, are scattered pigment cells, ("Melanoid.")

Dr. McWilliams, upon favorable report of the committee, was duly elected a member of the society.

### CHOLERA.

Prof. N. S. Davis resumed the discussion of cholera. He stated very clearly the effects of caloric upon the vital affinity of the tissues of the body, producing a laxity of tissue and a sluggish performance of the processes of nutrition and secretion which favor the invasion of disease. Added to these predisposing causes, is either the absence or the presence of something in the air, which by its action upon the excito-vascular nerves depresses their vitality, so that they cease to maintain that degree of vascular tonicity which is essential to health. Hence, exudation occurs in the form of perspiration upon the surface of the body, and in the form of a flux upon the mucous surface of the alimentary canal. As the disease advances the blood is drained of its watery portion until it becomes too thick to circulate, and the phenomena of collapse ensue. In explanation of the cases of speedy death where collapse occurs early, perhaps without vomiting and purging, Dr. Davis believed that the phenomena are referable to the overwhelming effects of a poison more than usually malignant, by which the vital affinity of the tissues is wholly arrested, producing death before the manifestation of the ordinary phenomena of the disease.

From these premises is to be deduced a treatment which shall serve to allay the irritability of the intestinal canal, to improve the tonicity and vitality of its coats and vessels, and to restrain the inordinate discharges from the blood. For this purpose Dr. Davis stated that in the stage of cholerine he was in the habit of prescribing the following mixture:

℞ Tr. opii,

Magnes. sulph.,

Acid sulph. arom., aa. ʒij.

Aquæ, ℥ij.

M. Dose, a teaspoonful every four hours. He also considered important the administration of mercurials, to correct the hepatic and renal secretions, and tonics to maintain the vitality of the tissues. Quinine should be avoided, because it is a nervous sedative, but strychnia would be found serviceable. Proper attention should be paid to diet and to drink. The practice of drinking between meals was objectionable, as also the use of alcoholic beverages. The free use of the vegetable acids would be found productive of good during the warm weather of summer. In his own experience the Professor had found simple vinegar and water a most agreeable and salutary drink.

Dr. Wickersham expressed his inability to find a theory of cholera that was completely satisfactory.

Dr. Marguerat stated that the observations which he had found recorded could not all be reconciled with the theories which had been presented to the society. It was a matter which demanded further investigation.

Dr. Lyman addressed the society, in support of the theory that cholera is the result of a morbid change produced in the watery portion of the blood, and that the phenomena of the disease are the efforts of nature to exclude this vitiated fluid from the tissues, and to eject it wholly from the system. Consequently, the proper course of treatment is that which, while upholding the powers of the sufferer, tends to favor rather than to counteract the eliminative process. In accordance with this view the speaker condemned the use of opiates, astringents and stimulants, which can only add to the danger of the patient by impeding natural excretion, by restraining the elimination of the *materies morbi*, and by favoring its circulation among the already enfeebled tissues.

He recommended moderate warmth and quiet, to support the vitality of the system; diaphoretics, emetics and even laxatives in cases where the eliminative efforts seemed to be impeded; mercurials in the early stage, to promote excretion; chloroform to relax spasm; and above all the free use of carbonic acid water, containing a small proportion of salt, to assist in the renewal of a healthy serum.

He also dwelt upon the importance of discriminating between a vital effort which is beneficial, and the mechanical inconvenience or even danger which may result from the effort. While vomiting and purging undoubtedly are a *mechanical* source of danger to a patient, it is important to keep in mind the greater *vital* injury which must result from opposing the removal of the cause which excites such phenomena. If the blood could be speedily and effectually purified through the natural channels, a fatal result would be almost impossible, and the more alarming features of the disease would not appear. But while the ordinary excretory channels are impeded, the effort to arrest the vicarious process which is going on through the only remaining avenues of exit, can be fraught with nothing but danger to the patient.

In conclusion, the speaker presented a table of more than six thousand cases of cholera, treated in the hospitals of London and Paris, from which it appeared that the percentage of mortality increased in exact proportion to the activity of the efforts made to counteract the phenomena of the disease.

Dr. Davis opposed the idea that vomiting and purging could be anything but prejudicial to a cholera patient. He advocated the use of astringents, but admitted that the natural processes of elimination should be encouraged. He did not place much confidence in the evidence of statistics, on account of the fallacies to which they are liable. As for the idea that the phenomena of cholera indicate the efforts of nature to relieve itself of offending matter, he could see no evidence of the existence of such matter in the intestinal discharges, and consequently regarded them as merely the penalty suffered by the patient on account of some deviation from the conditions necessary to health.

Dr. Marguerat replied in defense of the statistics produced by Dr. Lyman, showing that they were of the most trustworthy character, having been taken by Dr. Ross from the records of the hospitals of London and Paris. The statistics most favorable to the proposed method of treatment by weak saline solutions administered freely with cold water, were obtained from the Greville Street Hospital in London, a cholera hospital where the worst forms of the disease were subjected to this treatment with the remarkable result of only 19 per cent of deaths.

